# Talent concentration and competitive imbalance in European soccer

**DOI:** 10.3389/fspor.2023.1148122

**Published:** 2023-03-30

**Authors:** Bernd Frick, Tommy Kweku Quansah, Markus Lang

**Affiliations:** ^1^ Management Department, University of Paderborn, Paderborn, Germany; ^2^ Institute of Sport Sciences, University of Lausanne, Lausanne, Switzerland

**Keywords:** competitive balance, professional team sport, talent concentration, European soccer, player market values

## Abstract

**Introduction:**

While most of the available literature on competitive balance analyses its impact on ticket sales and TV audiences, less empirical research is available that examines the observable variation in competitive balance across leagues and over time. This paper studies the concentration of player talent and end-of-season league points to empirically assess whether leagues with a more equal distribution of player talent produce a more balanced competition than leagues with less equal distribution.

**Methods:**

The longitudinal data we use to estimate our empirical model comes from professional soccer leagues in twelve Western European countries from 2005/06 thru 2020/21, yielding 5,299 club-season observations.

**Results:**

Our empirical analysis indicates that talent concentration in a league significantly and positively impacts points concentration in that league. However, in specifications controlling for year, country, and division, this impact is only weakly significant or insignificant, suggesting that talent concentration does not significantly affect competitive balance in that league. Additionally, our findings demonstrate that the relationship between talent and points concentration does not vary considerably across the European leagues or over time.

**Discussion:**

Our results suggest that repeated participation in the UEFA Champions League, with its considerable monetary returns by (more or less) the same subset of teams, does not increase competitive imbalance in the respective national league. Thus, with relatively few additional regulatory interventions, the promotion and relegation system in the open European soccer leagues seems effective in ensuring a balanced competition.

## 1. Introduction

At the end of the 2018/19 season, the finalists of the UEFA Champions League (UCL) and the UEFA Europa League all came from the English Premier League (“EPL”). Since the EPL is the commercially most successful league and known to pay the highest player salaries, the press, and the public were quick to agree that (i) competitive balance in European soccer is at risk (1), (ii) on-field success is increasingly determined by money (2, 3), with smaller teams being priced out (4), and (iii) this trend is likely to continue and even to intensify (1). One year later, when the finalists of the two soccer competitions were teams from Germany, France, Spain, and Italy, with no English team among the final four, the claims muted for a while before picking up again in the season 2020/21, when three of the four finalists were again teams from the EPL (5–7).

Apart from the financial imbalance between the European soccer leagues, often symbolized by the distinction between the “Big-5” (i.e., the top five soccer leagues in Europe, which include the *Premier League* in England, the *Bundesliga* in Germany, *La Liga* in Spain, *Serie A* in Italy, and *Ligue 1* in France) and the remaining European leagues (8), growing economic and sporting imbalances between the clubs within the same league have been identified by some researchers (9–12), and contested by others (13–16).

While the majority of the available studies on competitive balance have used various measures to analyze its impact on sports demand (17–20), few studies have used competitive balance measures as the dependent variable, most notably investigating the impact of revenue sharing (i.e., media rights distribution, parachute payments) on the competitive balance of a league (21–25). This approach is limited as the analysis is typically restricted to one or a few leagues where the revenue composition varies across the clubs. Moreover, differences in the clubs' goals (win vs. profit maximization) need to be considered (26).

In this study, we take a different approach by analyzing whether leagues with a more equal distribution of player talent produce a more balanced competition than leagues with a less equal talent distribution. We use the concentration of player market values in selected European soccer leagues as a proxy of talent concentration across clubs. In our empirical analysis, we find that the level of talent concentration in a league has a weak and, in most specifications, statistically insignificant impact on the points concentration in this league, suggesting that the concentration of talent in a league leaves that league's competitive balance more or less unaffected. Moreover, we also find that this weak correlation between talent and points concentration does not vary a lot across the European leagues nor over time, suggesting that repeated participation in the UCL with its considerable monetary returns by (more or less) the same subset of teams does not increase competitive imbalance in the respective national league. Thus, our study extends the available research on competitive balance by empirically examining differences between leagues regarding the distribution of sporting talent and its consequences for seasonal competitive balance.

The remainder of the paper is organized as follows: Section 2 presents an overview of the literature on player market values, talent distribution, and competitive balance. Section 3 describes the dataset and the empirical model. Section 4 presents the results of the empirical investigation. Section 5 provides a discussion of our main findings, and section 6 concludes with a discussion of the limitations of our study and implications for future research.

## 2. Related literature

We have structured the literature review in the following manner: Firstly, we offer a concise overview of the measures utilized to quantify competitive balance. Next, we summarize the literature highlighting the importance of competitive balance for ticket and TV demand, as documented in the sports economics literature. Lastly, we analyze the literature on player market values and talent distribution across leagues and clubs.

### 2.1. Competitive balance in the sports economics literature

Rottenberg (27) stated that “the nature of the industry is such that competitors must be of approximately equal “size” if any are to be successful; this seems to be a unique attribute of professional competitive sports.” This argument was later picked up by (28), emphasizing the “first peculiarity of the economics of professional sports is that receipts depend upon competition among the […] teams, not upon business competition among the […] contenders, for the greater the economic collusion and the more the sporting competition the greater the profits”.

Building on these arguments, Cairns et al. (17) were the first to distinguish between short-, medium- and long-term competitive balance. Short-run competitive balance or game uncertainty (29) deals with the uncertainty surrounding a particular sporting event, such as a soccer match, while medium-term competitive balance focuses on within-season uncertainty. Long-run competitive balance captures the distribution of championships over time, i.e., domination by one team only or a few teams.

Over time, several measures have been developed to account for short-, medium- and long-term competitive balance. Each measure has specific strengths and weaknesses, which are unavoidable when describing a complex phenomenon with one summary measure (14). In our study, we are particularly interested in (changes in) medium-term competitive balance contingent on (changes in) talent concentration. Measures used to capture medium-term competitive balance are—among others—the dispersion of winning percentages, the Gini coefficient (*G*), the coefficient of variation (CoV), the concentration ratio, the distance to competitive balance, the relative deviation from the mean, the Theil Index, as well as the Herfindahl Index. In the end, as Penn and Berridge (14) put it, there is no “Holy Grail” in the measures characterizing within-season competitive balance because no single measure can be considered the correct or the most appropriate one in every circumstance. Each measure focuses on a different feature. The pros and cons of various measures of competitive balance in professional team sports are discussed in more detail by, for example, Humphreys (30), Utt and Fort (31), Fort and Maxcy (32) and Owen et al. (33).

According to Fort and Maxcy (32), the large and growing body of literature on competitive balance can be divided into two distinct streams: the literature analyzing levels of and changes in competitive balance (ACB) and the literature testing the uncertainty of outcome hypothesis (UOH). While competitive balance is an ex-post construct based on end-of-season league tables, outcome uncertainty is an ex-ante concept assessing probabilities of game or seasonal outcomes in advance. The ACB literature focuses on the analysis of competitive balance as such, from a time perspective or as a consequence of changes in league structures or mechanisms. The UOH literature analyses the impact of competitive balance on stadium attendance and/or TV viewership. In summary, “ACB aims at tracking (competitive) balance itself,” while “UOH is aimed at measuring fan welfare” [(32), p. 157].

The ACB literature that analyses the determinants of competitive balance is relatively scarce and relies mainly on a game-theoretical perspective. A particular focus of this stream of literature has been on the impact of revenue sharing on competitive balance. As the relationship between these two variables depends on many factors (such as the clubs' objectives, the specific sharing arrangements, the specifications of the revenue functions as well as the supply of talent), the findings reported in the literature are inconsistent (34, 35).

From an empirical perspective, Andreff and Bourg (36) compared pooled and individual club ownership of broadcasting rights and their influence on competitive balance across 16 European leagues. They conclude that the broadcasting rights redistribution mechanisms in French and English first-tier soccer in the 1990s not only improved competitive balance within the leagues but also promoted the clubs' incentives to win and invest in playing talent, as broadcasting revenues are determined by the individual clubs' ranking and their number of television appearances. Using a large dataset from 12 major European soccer leagues and covering a period of twenty-five years (1976–2000), Frick and Prinz (37) find that a more or less equal redistribution of the revenues earned through the collective sale of broadcasting rights (which may account for up to 50% of the teams' budgets) leaves the survival probabilities of recently promoted teams completely unaffected.

Wilson et al. (25) examined the impact of parachute payments on competitive balance in the English *Championship*. They found that an increase in the number of clubs with parachute payments and the overall value of these payments coincides with a reduction in competitive balance. Other authors have looked at the impact of UEFA's Financial Fair Play regulations (38–40) and the impact of financial inequality on competitive balance (41, 42)—resulting in no unanimous conclusion.

Several recent papers have examined the determinants of competitive balance in European soccer. For example, Scelles et al. (43) explore the determinants of competitive balance in European men's club soccer from 2006 to 2018. They propose a theoretical framework that includes seven additional variables to explain the drawing power of a league, the revenue distribution between and within leagues, and talent distribution. The results show that GDP significantly impacts competitive balance, while attendance from the previous year has no significant effect. Moreover, Gasparetto et al. (44) examine the factors influencing competitive balance in 22 of Europe's top-tier soccer leagues from 2004 to 2021. The study found that play-offs for relegation, average age, talent concentration, and standard deviation of team values harm competitive balance. In contrast, the number of teams in the league, Elo rank, local currency to Euro rate, and Gini index have a positive effect. In a similar study, Rappai and Fűrész (45) examine the relationship between player value, talent, number of superstars, and sports performance regarding competitive balance in the top five European soccer leagues.

Gasparetto et al. (44) and Rappai and Fűrész (45) have conducted studies closely related to ours, examining the factors influencing competitive balance in European soccer. In contrast, while Scelles et al. (43) include talent distribution in their theoretical framework, they do not directly test its impact. However, Gasparetto et al. (44) and Rappai and Fűrész (45) test talent concentration in the same manner as our study, and their findings indicate a positive relationship between talent concentration and competitive imbalance, which contrasts with our main finding.

Given the mixed results reported in the above ACB literature that examines the determinants of competitive balance and the correlation of the explanatory variables with other factors such as club objectives, league structure, and sharing arrangements, we conjecture that taking a step back by looking at the link between sports talent concentration and competitive balance is a promising empirical approach to align the conflicting results reported in previous studies.

### 2.2. Player market values and talent distribution across leagues and clubs

Sports fans are typically attracted by the absolute and relative quality of leagues and games and, ultimately, by the playing talent under contract (46). The absolute quality of a league can be approached by total league revenues, i.e., the sum of the revenues of the individual clubs (47). Game theory suggests that in an open league with a flexible talent supply, absolute quality is affected by talent investment and allocation (48). To increase the playing strength of their team and weaken their opponents, the managers of a few wealthy clubs may be tempted to sign “too many” talented players and bench some of them, so some top players are misallocated (47). The relative quality of a league refers to the talent distribution between the clubs in a league. Some studies have analyzed the effect of revenue-sharing arrangements on talent concentration (47, 49). In an open, win-maximizing league, revenue-sharing arrangements with net transfers from large-budget to small-budget clubs result in a more balanced distribution of talent (49), while total talent investment increases (47) because small-budget teams invest more than the large-budget teams reduce.

In soccer, Flores et al. (50) contend that competitive imbalance may arise from ability gaps between the top tier and the remainder of recruited players, mainly when the talent pool is small, for example, when the eligible population is small. Conversely, when the talent pool is large, such as in more populated countries, ability gaps are less pronounced, resulting in an improved competitive balance. In a global marketplace, soccer talent can be bought and sold worldwide, following the simple rules of supply and demand. If a player with given talent is paid less by his current club than he is worth to other clubs, he will be signed by another club, where his marginal product is expected to be higher—given that markets are efficient (16). In an investigation of competitive balance in professional baseball in the years 1901 to 2000, Schmidt and Berri (51) observed that the level of competitive balance in *Major League Baseball* (MLB) has—in contrast to the prevailing views of sports insiders and sports media—improved after 1960 not because of institutional changes but due to an increase in the size of the talent pool. Similarly, Schmidt (52) attributes the improved competitive balance in baseball to increases in the population of players that MLB can employ due to player immigration.

Quansah et al. (53) define a player's market value as a theoretical construct that approximates the current market price for releasing that player from an existing contract, irrespective of the remaining contract length. That value is determined by individual characteristics, such as the player's age, position, and past performance, club characteristics, such as market size and (historical) performance, as well as prevailing market conditions (54).

Using data from www.transfermarkt.de, Herm et al. (55) find that in a sample of 67 player transfers occurring during the winter break 2011/12 in the German *Bundesliga*, the market values explain almost entirely (*R*^2^*^ ^*= 0.90) the variance in the paid transfer fees. Peeters (56) finds in a sample of more than 1,000 qualifying matches and World Cup/Euro Cup matches over the period 2008 to 2014 that forecasts of match results based on the crowds' evaluations are far more accurate than standard predictors such as the FIFA ranking or the ELO rating of the two opposing teams. Using data from ten consecutive seasons from 2006 thru 2015 in *Major League Soccer*, Prockl and Frick (56) find that player market values and salaries are highly correlated at +0.75, a finding that has recently been confirmed by Frick and Winner (58) using data from one season in the German *Bundesliga* (2014/15) and the Italian *Serie A* (2015/16). Thus, we conjecture that differences in team values explain differences in performance. More specifically, we expect a larger concentration of playing talent to lead to less competitive balance.

## 3. Methods

### 3.1. Data

Our starting dataset consisted of 5,299 club-season observations from professional soccer leagues in twelve Western European countries from 2005/06 thru 2020/21. It included the market value of each squad at the beginning of the respective season and the clubs' final league position at the end of that season. The first season for which the market values of the squads are available is 2006/07 (except for the German teams, where the market values are already available for 2005/06).

While a club's wage bill has been previously used as an indicator of player quality and as a predictor of team performance (59, 60), this approach is limited by the fact that salaries are fixed for the length of a contract (54) and do not reflect longer periods of players' form highs or lows, nor injuries. To overcome these limitations, the present study instead employs the market value of a squad as an indicator of its absolute quality. Researchers widely use market values at both the individual and team levels as a proxy [e.g., (61–64)].

The market values we use in this study have been retrieved from www.transfermarkt.de, a crowd-driven online platform whose registered users discuss and express their opinions about, among other things, the market values of players in designated forums. It was founded in Germany in 2000 and is now available in eight languages; the English version, for example, was added in 2009. The portal offers different levels of participation, the most exclusive being the discussion of market values, participation in the so-called “rumor mill,” and surveys dedicated to specific football-related topics. A user is admitted to the exclusive areas only after s/he has published a minimum of 100 qualitative posts, which leads to promotion to the status of an “expert.” After reaching a certain level of blog activity, individual users can also apply for leadership positions such as e.g., data scout or godfather.

Transfermarkt.de is selective because player values are not simply calculated as the mean (or the median) of the individuals' suggestions. Instead, a particularly empowered community member—a “judge”—chooses to aggregate the information provided by the community on a case-by-case basis, implying that s/he is entitled to reduce the impact of values s/he considers “outliers” or even completely delete these. Thus, the judge performs the complex task of filtering, weighting, and aggregating information by viewing the source of information (a person with a limited number of suggestions vs. an experienced community member with hundreds of suggestions) as well as the reason(s) provided as justification(s) for specific estimates (e.g., only one or two-player characteristics vs. a lengthy description of that player's abilities).

Whereas using crowd-sourced data is not without criticism, several studies have found strong correlations between crowd-sourced market values on transfermarkt.de and actual transfer fees (55, 62, 65). Critics have raised objections about the potential for manipulation, social influence, and knowledge deficiencies among community members (66, 67), as well as concerns about the objectivity and efficiency of the data (68). Nevertheless, the results of this study align with previous findings that suggest the use of crowd-sourced market values can provide a valuable indication of a squad’s absolute quality.

Table 1 presents the composition of our dataset with 285 league-season observations.

**Table 1:** Composition of the dataset.

Country	Divisions and number of teams	Number of observations
Austria	1^st^ Division: 12 (since 2018/19; 10 before)	156
Belgium	1^st^ Division: 18 (2006/07–2008/09)	248
	16 (2009/10–2019/20)	
	18 (since 2020/21)	
England	1^st^ Division: 20	300
	2nd Division: 24	360
	3rd Division: 24 (23 clubs in 2019/20)	359
France	1^st^ Division: 20	300
	2nd Division: 20	300
Germany	1^st^ Division: 18	288
	2nd Division: 18	288
	3rd Division: 20 (inaugural season 2008/09)	260
Greece	1^st^ Division: 16 (2006/07–2012/13)	240
	18 (2013/14–2014/15)	
	16 (2015/16–2018/19)	
	14 (since 2019/20)	
Italy	1^st^ Division: 20	300
	2nd Division: 22 (2006/07–2017/18)	323
	19 (2018/19)	
	20 (since 2019/20)	
Netherlands	1^st^ Division: 18	270
Portugal	1^st^ Division: 16 (2006/07–2013/14)	254
	18 (since 2014/15)	
Spain	1^st^ Division: 20	300
	2nd Division: 22	330
Switzerland	1^st^ Division: 10	150
Turkey	1^st^ Division: 18 (21 in 2020/21; no relegation)	273

The table shows that the number of clubs in each league varies considerably between the twelve European soccer leagues under consideration. For example, Switzerland has the lowest number of clubs, with only ten teams playing in the first division, whereas the English second and third division counts 24 clubs each.

### 3.2. Model and descriptive statistics

To provide an accurate picture of the concentration of playing talent on the one hand and the competitive balance of a league on the other hand, we calculated, for each season in each of the leagues, the Gini coefficient of the clubs' player market values and the Gini coefficient of the number of end-of-season league points. The traditional Gini coefficient can take any value between zero and one, where a coefficient of zero denotes perfect equality, and a Gini coefficient of one indicates maximal inequality among values. However, these maximal values are not to be expected in soccer.

Consider the concentration of end-of-season league points: a Gini coefficient of one would imply that at the end of the season, one club has won all its matches, while all other clubs have lost all their matches. This outcome is impossible to occur, and thus a value close to one is not to be expected. The same logic applies to the concentration of playing talent: Even the worst teams in a league will have some playing talent under contract (e.g., their market value will be larger than zero) and, therefore, values of the Gini coefficient close to one are again not to be expected.

The maximum value of the Gini coefficient for the end-of-season league points depends on the number of clubs in a league. For example, in a league with 20 clubs, the maximum of Gini coefficient is 0.350 (when the champion wins all its 38 matches, the runner-up wins 36 matches and loses the two against the champion, while the last team loses all its 38 matches, and the second-last team wins its two matches against the last team). The maximum of the Gini coefficient varies with league size, but the variations are tiny.

It is worth noting that the optimal constellation for maximizing a competitive balance measure highly depends on the specific measure being used. The considerations mentioned above are specific to the Gini coefficient. For instance, the DCB reaches its maximum value of one in a 20-club league where the top seven teams win their games, and the remaining 13 teams draw. Additional details can be found in Avila-Cano et al. (69), who identify the maximum concentration of results for different sports competitions.

In the predominantly US literature, the limited range of the Gini coefficient has been criticized, as well as the fact that teams in the US do not play balanced schedules. However, the latter is not valid for European soccer leagues, which mostly are played as round-robin tournaments (each team plays each other team once at home and once away). Considering the limited range, we use the Coefficient of Variation (CoV) of points as a second measure. The correlation between the Gini of the number of points and the CoV of points is exceptionally high at *r* = 0.962. A novel and alternative measure that accounts for the number of teams in a league and the point system in soccer, where a win is worth three points, and a draw is worth one point, is the “distance to competitive balance” measure proposed by Triguero Ruiz and Avila-Cano (70).

Table 2 presents the descriptive statistics for all main variables included in the analysis (the distribution of points and talent concentration is displayed in Figure A1 in the Supplementary Material).

**Table 2:** Descriptive statistics (*n* = 285 league-season-observations, 2005/06 thru 2020/21).

Variable	Mean	Std. Dev.	Min.	Max.
Gini of Points	0.158	0.034	0.072	0.244
CoV of Points	0.298	0.067	0.129	0.545
Gini Talent	0.343	0.103	0.066	0.611
Austria	0.053	—	0	1
Belgium	0.053	—	0	1
England	0.158	—	0	1
France	0.105	—	0	1
Germany	0.158	—	0	1
Greece	0.053	—	0	1
Italy	0.105	—	0	1
Netherlands	0.053	—	0	1
Portugal	0.053	—	0	1
Spain	0.105	—	0	1
Switzerland	0.053	—	0	1
Turkey	0.053	—	0	1
Division 1	0.635	—	0	1
Division 2	0.267	—	0	1
Division 3	0.098	—	0	1

It appears from Table 2 that the average Gini of points *G_P_* is 0.158, and the average Gini of talent concentration *G_T_* is 0.343. Moreover, Table 2 shows that the minimum value of the Gini of points is 0.072 (second division in Spain in season 2013/14), and the maximum is 0.244 (first division in Greece in 2019/20). On the other hand, the concentration of playing talent is considerably higher yet similar to the concentration of earnings of full-time employees in most Western European countries (0.340). Here we observe a relatively large dispersion with a minimum of 0.066 (third division in Germany in season 2015/16) and a maximum of 0.611 (first division in Portugal in 2019/20). The average CoV of points is 0.298, the minimum value is 0.129 (second division in Spain in 2013/14), and the maximum is 0.545 (first division in Switzerland in 2011/12).

## 4. Results

This section presents our results and is structured as follows: First, we examine the average degree of talent and points concentration in the 12 soccer leagues under consideration. Second, we analyze the evolution of talent and points concentration in the Big-5 leagues over time. Third, we empirically examine the relationship between talent concentration and points concentration using a linear regression model.

### 4.1. Talent and points concentration by country and division

**Figure 1:**
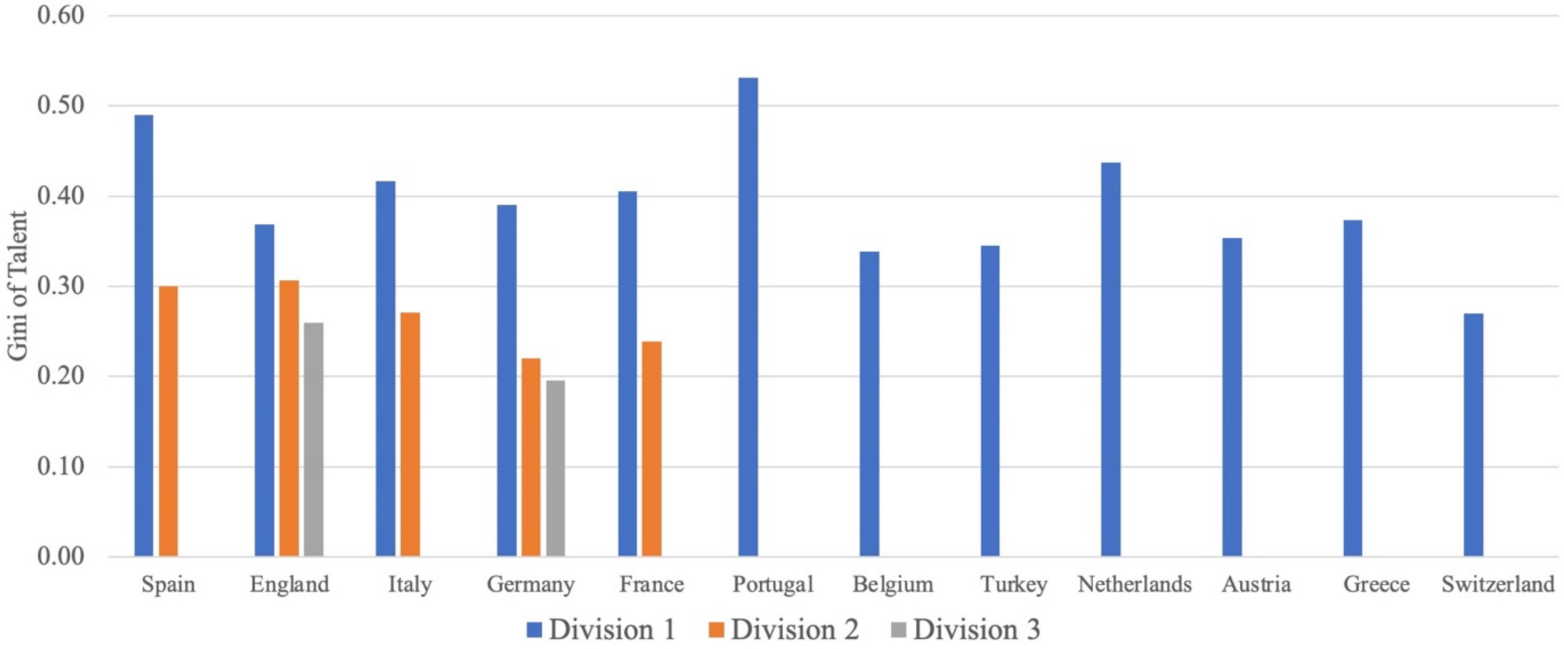
Talent concentration by country and division.

Figure 1 displays the degree of talent concentration in the twelve soccer leagues averaged over the observation period. The blue bars depict the first divisions, the orange bars are the second divisions, and the grey bars are the third divisions. Recall that a Gini coefficient of zero would indicate a perfectly equal distribution of playing talent in a league. In contrast, a coefficient of one would represent perfect inequality, where one team in the league has all the talent while all other teams have no talent.

Figure 1 shows that the *Primeira Liga* in Portugal has the highest level of talent concentration with 0.525, followed by *La Liga* in Spain with 0.490 and *Eredivisie* in the Netherlands with 0.425. The lowest level of talent concentration (0.275) can be found in the Swiss *Super League*. Moreover, it appears from Figure 1 that the second and third divisions are characterized by a notably lower degree of talent concentration than the respective first divisions.

**Figure 2:**
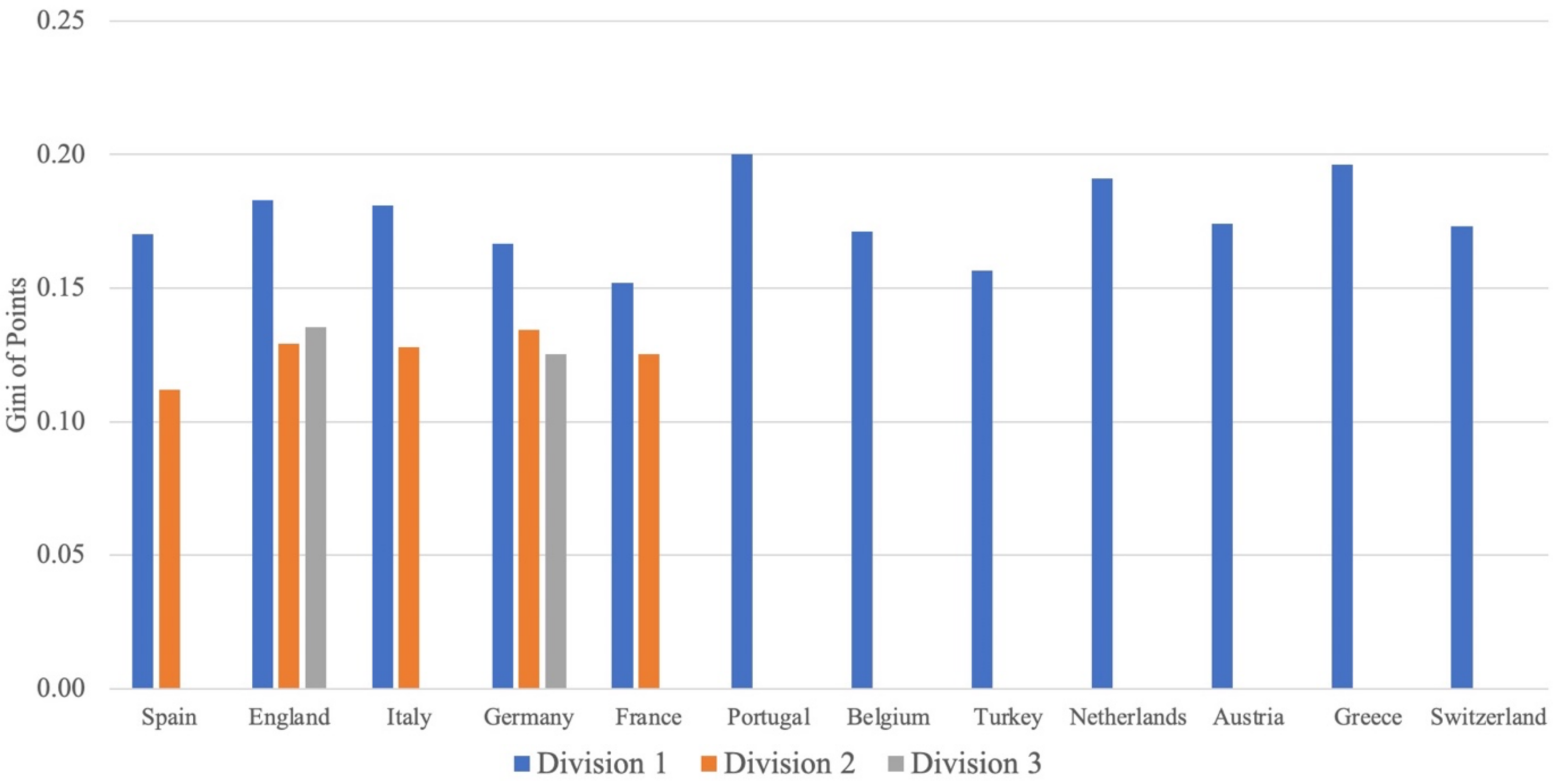
Gini of points concentration by country and division.

Next, we examine the level of competitive (im-)balance measured by our Gini of points concentration. Figure 2 illustrates the Gini of points concentration in the 12 leagues averaged over the years. The differently colored bars again represent different divisions (blue bars = first divisions, orange bars = second divisions, grey bars = third divisions). For the corresponding figure with CoV of points concentration instead of Gini of points concentration, see Figure A2 in the Supplementary Material.

Recall that the Gini of points concentration has a somewhat limited range compared to the talent concentration measure. For example, in a league with 20 clubs, it can only take values between zero and 0.350. Thus, a coefficient of zero would indicate a perfectly equal distribution of final points, whereas a coefficient of 0.35 would represent perfect inequality in such a league.

Among the first divisions, we find the *Primeira Liga* in Portugal to be the least balanced league with a points concentration of 0.2, while the French *Ligue 1* turns out to be the most balanced league with a Gini of 0.15. Among the second divisions, Spain has the most balanced and Germany has the least balanced league. Finally, Germany's third division is slightly more balanced than England's League One. Overall, the lower divisions are more balanced than the respective higher division. Thus, one might think that the European leagues are characterized by a relatively high level of competitive balance, with a value of 0.2 for the least balanced league. However, due to the limited range of the Gini of points, a value of 0.2 indicates a relatively high concentration level.

We computed the correlation between these two variables to gain insights into the relationship between talent concentration and points concentration across different countries. The results of our analysis are presented in Table 3.

**Table 3:** Correlation gini talent and gini points by country (only first divisions).

Country	Correlation
Austria	−0.04
Belgium	−0.460
England	0.286
France	0.588
Germany	0.381
Greece	0.412
Italy	0.326
Netherlands	−0.108
Portugal	−0.259
Spain	0.561
Switzerland	−0.053
Turkey	−0.267

One interesting finding that emerged from Table 3 is that the correlation between talent concentration and points concentration was positive for all the Big-5 leagues, which suggests that in these leagues, there is a positive relationship between the concentration of talent in a few top teams and the distribution of points across the league. On the other hand, in the rest of the leagues that we analyzed (apart from Greece), we found a negative correlation between talent concentration and points concentration. This result may indicate that in these leagues, a few top teams with a high concentration of talent do not necessarily dominate the league and accrue a disproportionate share of the points. Instead, the points are distributed more evenly across the league, with more teams competing at a similar level.

Before proceeding to the regression analysis, where we estimate the impact of talent concentration on the concentration of points, we use the longitudinal nature of our data set to identify potential time trends.

### 4.2. Evolution of talent and points concentration in the Big-5 Leagues

**Figure 3:**
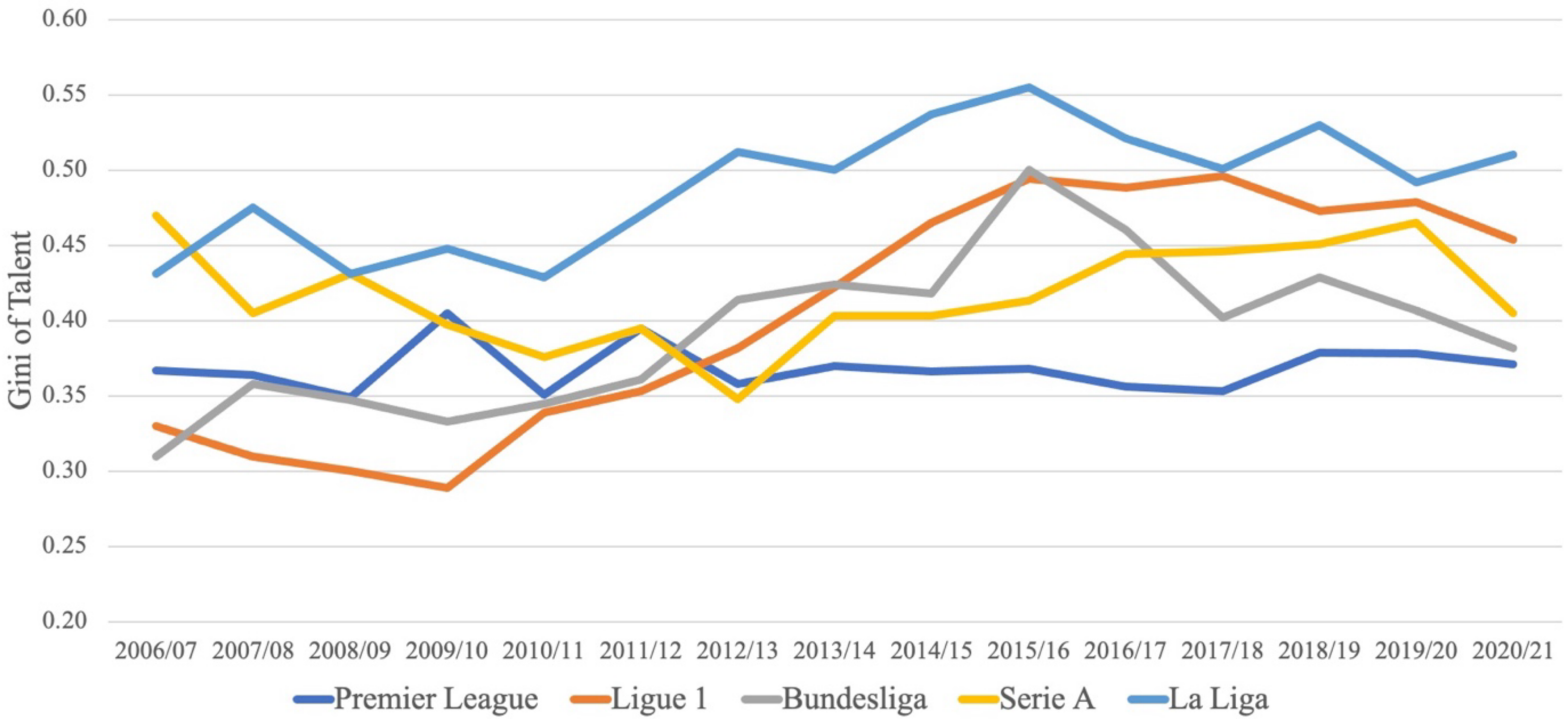
Evolution of talent concentration in Big-5 leagues.

This section examines the evolution of points and talent concentration over time. Here we restrict our attention to the Big-5 leagues and their respective first division. Figure 3 displays the Gini of talent concentration in the Big-5 leagues between 2006/07 and 2020/21.

The evolution of talent concentration has been quite heterogeneous in the Big-5 leagues over the last 15 years. Talent concentration has largely increased in the French *Ligue 1* since the 2009/10 season and has been relatively stable in the English *Premier League*. Interestingly, the Italian *Serie A* shows a u-shaped development of talent concentration over time, whereas the picture for the German *Bundesliga* resembles an inverted u-shape.

**Figure 4:**
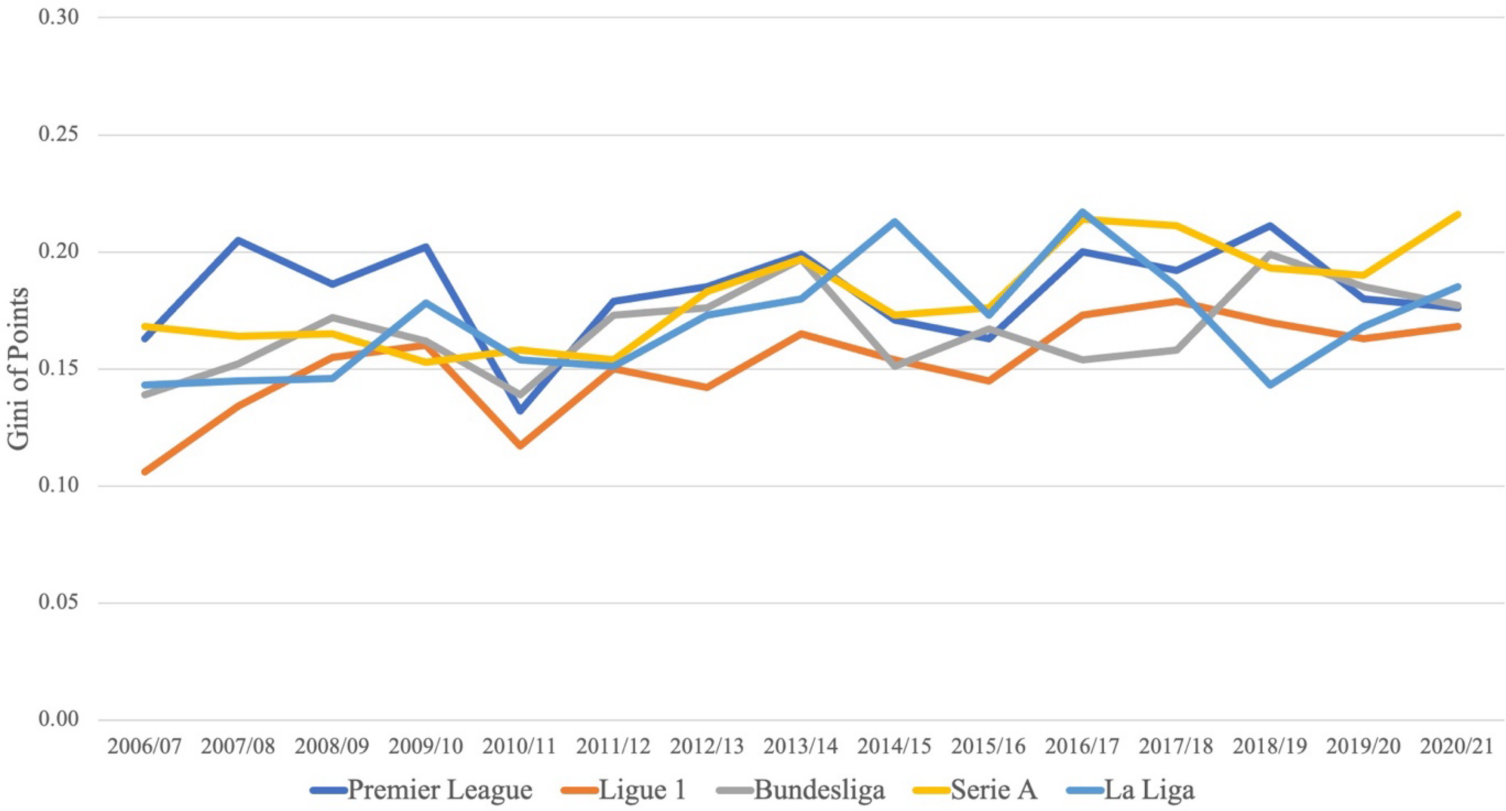
Evolution of points concentration in Big-5 leagues.

In Figure 4, we examine the evolution of the Gini of points in the Big-5 leagues during the observation period (Figure A3 in the Supplementary Material shows the corresponding figure with the evolution of CoV of points concentration in the Big-5 leagues).

Figure 4 shows quite some fluctuations concerning the level of points concentration in the Big-5 leagues between 2006/07 and 2020/21. There is, however, no discernible trend in any of the five leagues, suggesting that competitive balance has remained relatively stable over the last 15 years.

### 4.3. Measuring the relationship between talent concentration and points concentration

We now examine the relationship between talent concentration and points concentration to check whether leagues with a high level of talent concentration are characterized by a high level of imbalance in terms of points concentration.

**Figure 5:**
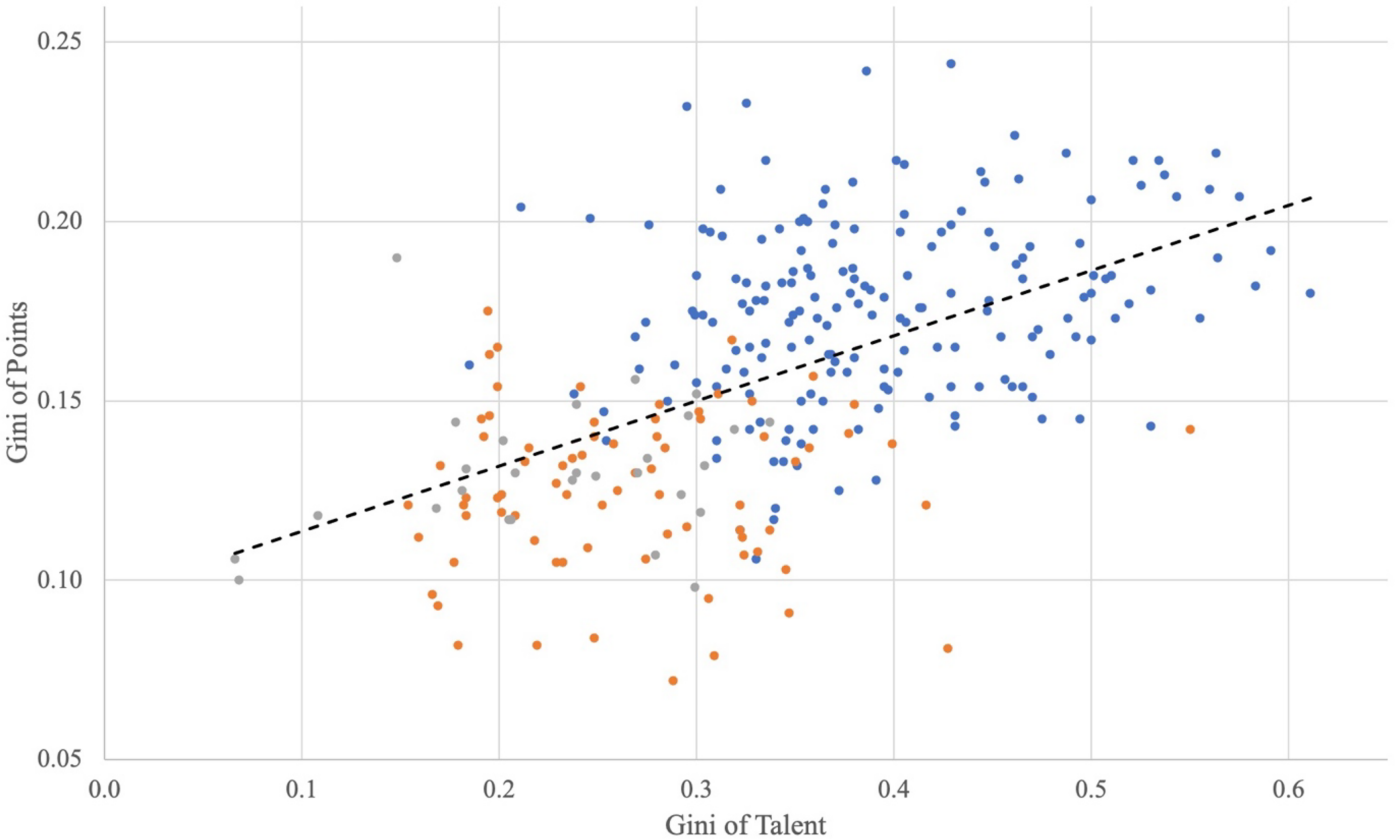
Talent and points concentration.

First, we visualize the relationship between talent concentration and the Gini of points concentration in a scatterplot (Figure 5). Each dot represents the level of talent and points concentration in a particular league in a specific season. The blue dots represent first-division clubs, while the orange and grey dots display second and third-division clubs. The black dashed line illustrates the regression line (the corresponding Figure A4 with CoV of points can be found in the Supplementary Material).

It is essential to mention that the outliers we observe in the scatterplot are not systematic, i.e., no single league drives this result. In addition, the regression line is upward-sloping and thus implies a positive correlation between talent and points concentration. In other words, the more unequal talent is distributed, the lower the competitive balance in this league.

This positive correlation between talent and points concentration is confirmed by our regression results in Table 4 below. The table presents the estimation results where the dependent variable (points concentration) is measured by the Gini coefficient (Panel a) and by the Coefficient of Variation (Panel b) of talent.

**Table 4:** Estimation Results

Panel a: Gini of points concentration				
Model	(1)	(2)	(3)	(4)
Dependent Variable	Gini of Points Concentration
Gini Talent	0.182***	0.186***	0.182***	0.039
Concentration	(0.020)	(0.002)	(0.023)	(0.022)
Year Dummies	No	Yes	Yes	Yes
Country Dummies	No	No	Yes	Yes
Division Dummies	No	No	No	Yes
Constant	0.095***	0.085***	0.098***	0.144***
	(0.008)	(0.006)	(0.001)	(0.008)
*N*	285	285	285	285
Adj R2*100	30.4	32.6	53.0	62.1
Panel b: Coefficient of Variation of points.				
Model	(1)	(2)	(3)	(4)
Dependent Variable	Coefficient of Variation of Points
Gini Talent	0.356***	0.367***	0.357***	0.087*
Concentration	(0.040)	(0.041)	(0.0248)	(0.042)
Year Dummies	No	Yes	Yes	Yes
Country Dummies	No	No	Yes	Yes
Division Dummies	No	No	No	Yes
Constant	0.176***	0.154***	0.186***	0.274***
	(0.017)	(0.012)	(0.018)	(0.014)
*N*	285	285	285	285
Adj R2*100	29.8	32.8	55.7	64.1

Standard errors (clustered at country level) in parentheses.

* *p* < 0.10, **p < 0.05, ***p < 0.01.

Without any further controls, our econometric analysis seems to confirm the hypothesis that the level of talent concentration in a league has a highly significant and positive impact on the concentration of points or the coefficient of variation of points in this league as an increase of talent concentration leads to an increase in the concentration of end-of-the-season league points. In other words, the degree of talent concentration seems to be a major driver of competitive balance in a league.

The results do not change qualitatively using alternative measures such as the Theil Index or the Herfindahl Index. Moreover, controlling for the number of teams promoted and relegated each season relative to the number of teams in a league leaves our findings unaffected. This result is perhaps surprising as a larger number of promotions may induce new teams to invest less in the quality of their squads as they anticipate being relegated again—with the consequence that talent concentration increases.

Comparing models (1) to (4) in Panels (a) and (b) yields the following additional insights: In Model (2), we include year dummies to examine whether the correlation between talent and points concentration has changed over time. Our result indicates that this is not the case because the coefficients of talent concentration for both dependent variables (Gini of points and the CoV of points) in Model (2) are not any different from the ones in Model (1). Thus, the relationship between talent concentration and points concentration has been relatively stable over the years.

In Model (3), we include country dummies (together with year dummies) and again find that the coefficient of the talent Gini remains unaffected compared with Model (2). Like the above result, the positive correlation between talent and points concentration does not vary systematically across the European leagues.

Finally, we include division dummies in Model (4) in addition to the other dummy variables and find that the coefficient of our talent concentration measure decreases significantly compared to Model (1) when the dependent variable is measured by the CoV of points (Panel b), indicating a weaker impact of talent concentration on points concentration. Talent concentration is no longer statistically significant when using the points Gini as the dependent variable (Panel a). These results are intuitive since lower divisions are more balanced than higher ones (see Figure 2). We obtain the same results if we restrict the analysis to the Big-5 leagues (see Table A1 in the Supplementary Material).

## 5. Discussion and conclusion

Our study focuses on talent concentration within and across European soccer leagues and its impact on seasonal competitive balance. We find that across the 12 first divisions and averaged over the observation period from 2005/06 thru 2020/21, the *Primeira Liga* in Portugal and *La Liga* in Spain have the most unequal talent distribution, while the Swiss *Super League* has the most equal talent distribution. However, the differences in talent concentration across the leagues are relatively small. Moreover, for countries where information on second and third-division teams is available, these divisions are characterized by a more equal talent distribution than the respective first division.

During the observation period (2005/06–2020/21), broadcasting and commercial rights of the UEFA Champions League (UCL) competition have witnessed a tremendous increase from €606 m in 2005/06 to €2,791 m in 2020/21 (71). Likewise, the prize money allocated among the participating teams has increased considerably. UCL winner 2020/21, Chelsea FC, generated an estimated €111 m in UCL price money alone—excluding the redistributed market pool and excluding match day revenues, which again add considerably to this amount (72, 73). Runner-up Manchester City earned €103 m in UCL prize money, while Borussia Dortmund, eliminated in the quarter-finals, still made an estimated €72 m in UCL prize money (73). The observed rise in broadcasting and commercial rights, as well as prize money allocated among the participating teams in the UCL, has caused concern about the potential negative impact on the competitive balance in European soccer (5–7). However, our research findings challenge this notion, suggesting that repeated UCL participation and its monetary returns do not necessarily lead to an increase in competitive imbalance within the domestic leagues. While the UCL may provide significant financial rewards for the participating teams, which account for a large share of the annual revenues even for the biggest clubs in Europe (74), our research indicates that other factors, such as revenue-sharing mechanisms and the promotion and relegation system might play a more critical role in determining competitive balance within a league. We acknowledge that our findings are promising, but it is essential to note that further research in this area is necessary. For instance, future studies could investigate the potential impact of UCL participation on the transfer market, as successful teams may be more attractive to higher-quality players, which could widen the gap between the richest and poorest clubs in Europe.

Our study adds to the ongoing debate about the UCL's impact on competitive balance in European soccer. Although it is clear that the competition provides significant financial rewards for the participating teams, it is also essential to consider the potential effects in the broader industry. Our research offers insights that could inform future discussions about maintaining a competitive and equitable soccer environment for all stakeholders.

Regarding competitive balance in the first divisions, we find that the Portuguese league is the least balanced, followed by the Greek and the Dutch leagues (these are relatively small countries with rather large leagues that have been dominated for many years by two or three teams). The most balanced league is the French *Ligue 1*. Again, the lower divisions are more balanced than the respective higher divisions. Like in the case of talent concentration, the differences across the leagues are relatively small. In sum, European soccer leagues are relatively homogeneous with respect to talent and points concentration.

Looking at the concentration of talent over time, it appears that it has increased in the French *Ligue 1* since 2009/10 and in the German *Bundesliga* as well as in the Spanish *La Liga* between 2006/07 and 2015/16, while it has been relatively stable in the English *Premier League*. Interestingly, the Italian *Serie A* shows a u-shaped development of talent concentration over time. Concerning points concentration in the Big-5 leagues, we see some fluctuation between 2006/07 and 2020/21, but we do not observe a significant trend (up or down) in either league.

As long as we do not add any further controls, our regression analysis seems to confirm recent studies such as Rappai and Fűrész (45) and Gasparetto et al. (44) that have shown statistically significant impact of talent concentration on the concentration of end-of-season league points—suggesting that leagues with a more unequal talent distribution are characterized by a lower level of competitive balance. However, since the significant impact of talent on points concentration is weaker for lower divisions, this effect disappears once we add division dummies to the estimations. Our results thus confirm earlier findings by Szymanski (15, 75), who observed a relatively stable competitive balance over time, despite increased financial inequality among clubs in English professional football. Finally, our results show that the impact of talent on points concentration does not vary much across the European leagues, nor over time (i.e., no time trend can be identified).

Our paper contributes to the literature on competitive balance in sports that primarily analyses the impact of competitive balance on sports attendance, while only limited empirical research explores the determinants of competitive balance.

Our study has important implications for league governing bodies in European soccer. Suppose the objective is to retain competitive balance. Our results suggest that with relatively few additional regulatory interventions, the promotion and relegation system in the open European soccer leagues effectively ensures a balanced league. The “punish the loser” (76) approach by relegating poorly performing teams and replacing them with the top-performing teams from the next lower division appears to be an effective mechanism. Although recently promoted teams have a disproportionate probability of being relegated again in European soccer leagues, new franchises in the North American major leagues typically struggle even more in their first year. Thus, the “liability of newness” is even more pronounced in North America than in Europe (77). In addition, the current system of promotion and relegation in European soccer leagues effectively promotes competitive balance without creating the perverse incentives of “losing to win” that are present in closed leagues operating with reverse-order drafts, such as the North American major leagues (78, 79).

Our study has a few limitations that should be addressed in future analyses. Firstly, we utilized the market values of teams at the start of their respective seasons to calculate talent Ginis. However, in certain countries, the transfer window remains open for a few weeks into the season, enabling clubs to sign additional players after the official start. Additionally, clubs can recruit mid-season during the winter transfer window, leading to different market values for those teams that remain active on the transfer market. It is crucial to investigate whether and to what extent this affects the results we obtain. Secondly, we purposely focused on leagues in Western European countries. However, it may be valuable to include leagues from Eastern and South-eastern Europe, such as Poland, Russia, Serbia, and Croatia, given that these countries' national associations score high in UEFA's and/or FIFA's rankings.

In conclusion, our study provides important insights into the relationship between player talent concentration and competitive balance in professional soccer leagues across twelve Western European countries. Our findings suggest that while the concentration of talent in a league positively impacts points concentration, this relationship is weak and insignificant when controlling for other factors. Moreover, our research suggests that participation in the UCL, with its considerable monetary rewards, does not significantly increase competitive imbalance in the domestic leagues. Our results have important implications for policymakers, soccer clubs, and fans concerned about maintaining competitive balance in the sport.

## Data Availability

Publicly available datasets were analyzed in this study. This data can be found here: https://www.transfermarkt.com.
