# Supplementary material for: Talent concentration and competitive imbalance in European soccer
Source: Front Sports Act Living. 2023 Mar 30;5:1148122. doi: 10.3389/fspor.2023.1148122 (PMC10097894; doi:10.3389/fspor.2023.1148122)
Supplement: Supplementary file 1 [file Datasheet1.docx]

**APPENDIX**

**Figure A1:** Distribution of Points and Talent Concentration


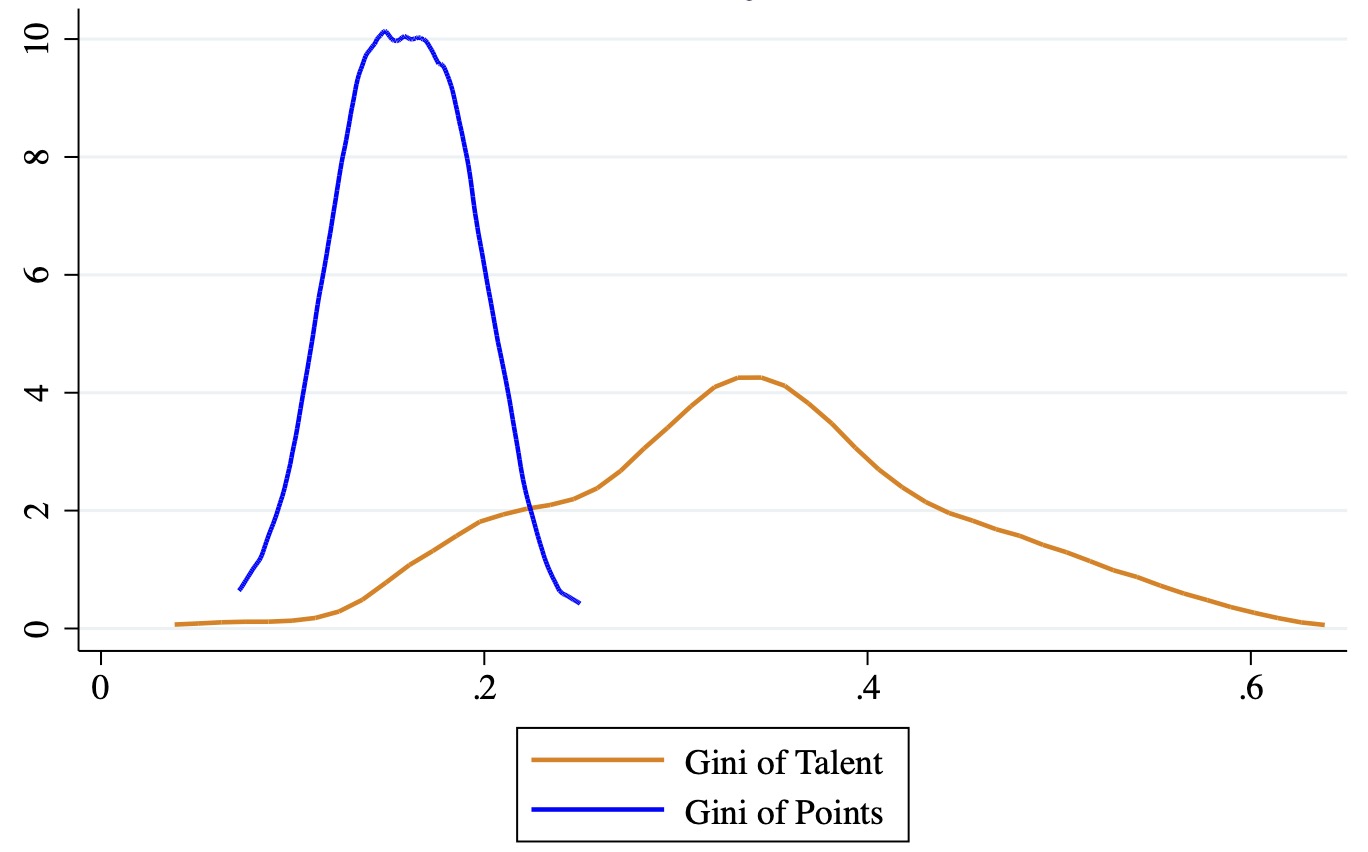


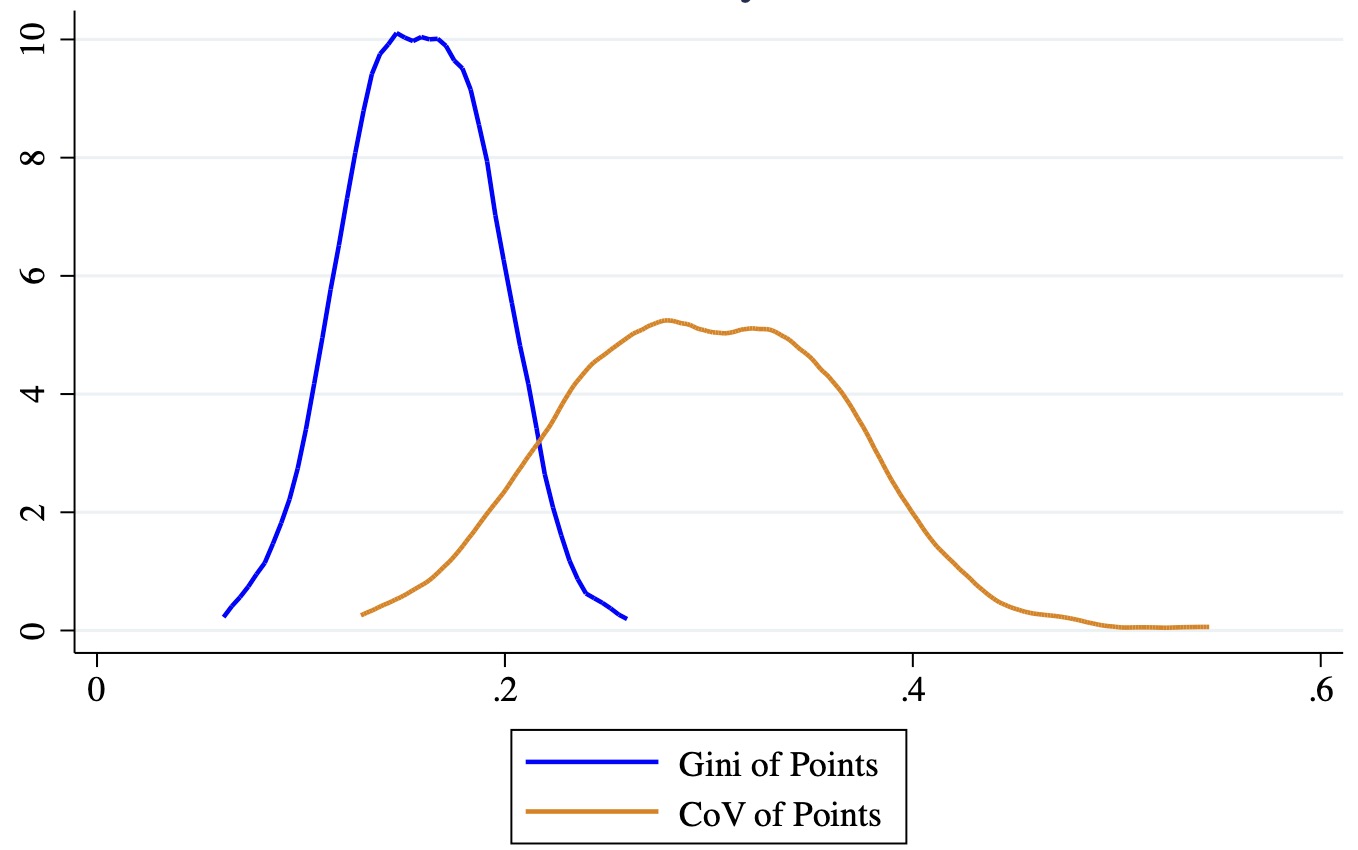


**Figure A2:** CoV of Points by Country and Division


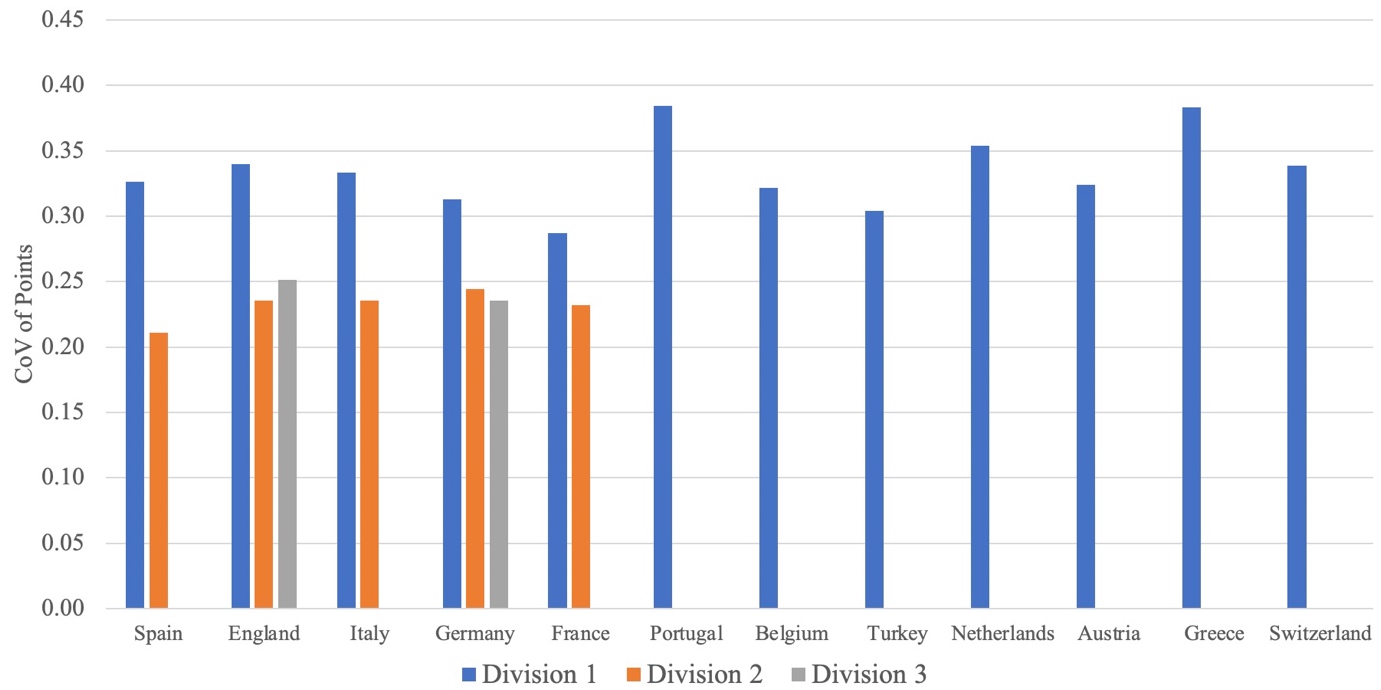


**Figure A3:** Evolution of CoV of Points in Big-5 Leagues


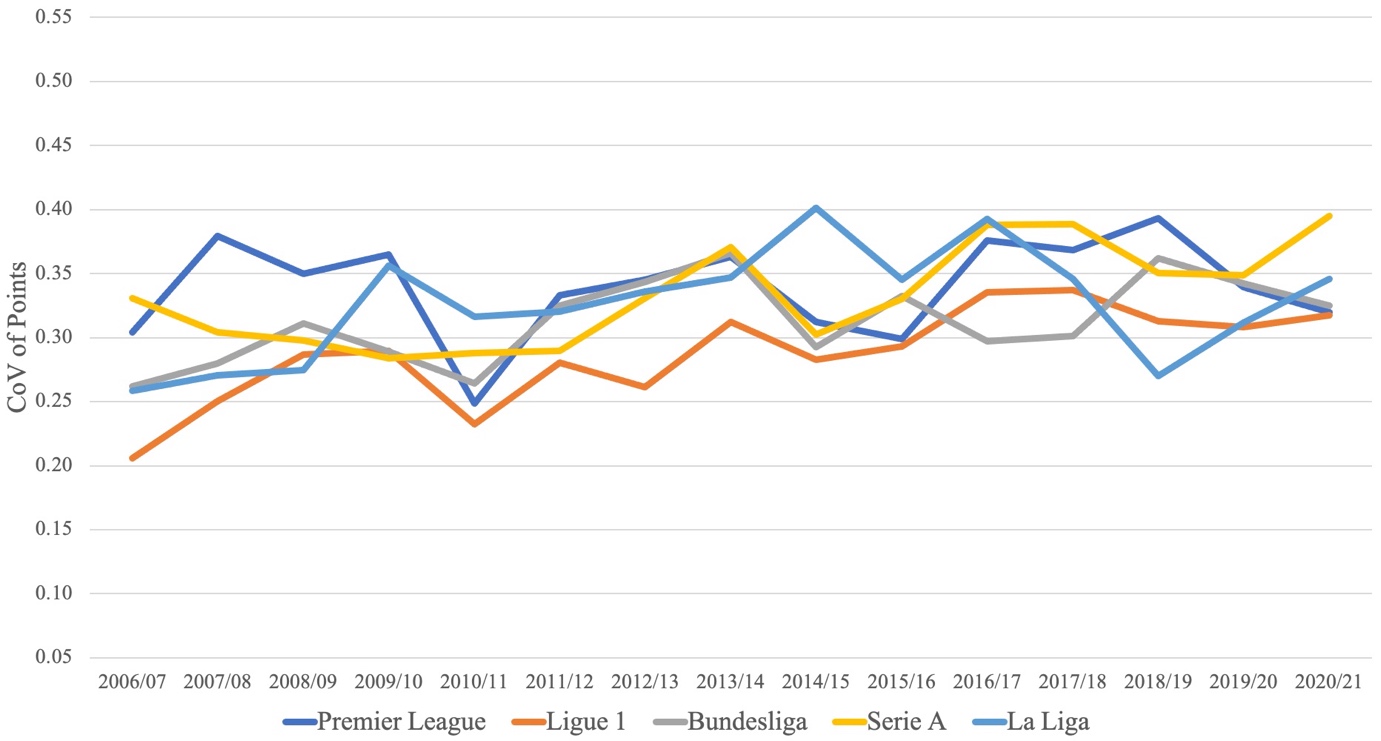


**Figure A4:** Talent Concentration and CoV of Points


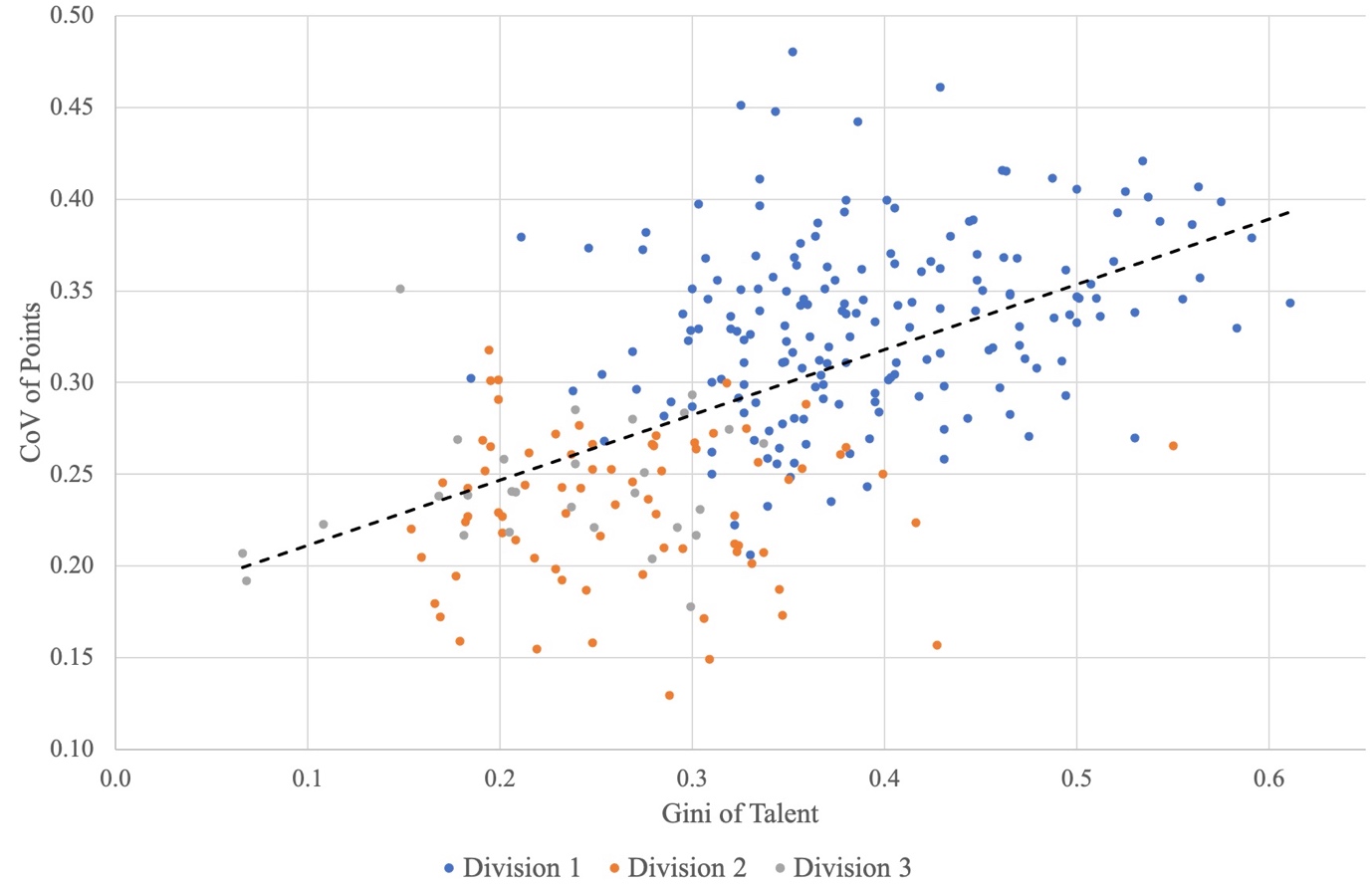


**Table A1:** Estimation Results for Big-5 Leagues

| Model | (1) | (2) |
| --- | --- | --- |
| Dependent Variable | Gini of Points | CoV of Points |
| Gini Talent | 0.0621 | 0.124^*^ |
|  | (0.0299) | (0.0534) |
| Constant | 0.144^***^ | 0.264^***^ |
|  | (0.0113) | (0.0209) |
| *N* | 180 | 180 |

Standard errors (clustered at country level) in parentheses

^*^ *p* < 0.10, ^**^ *p* < 0.05, ^***^ *p* < 0.01

**Table A2:** Estimation Results (controlling for the number of teams promoted and relegated each season)

| Model | (1) | (2) | (3) | (4) |
| --- | --- | --- | --- | --- |
| Dependent Variable | Gini of Points Concentration | | | |
| Gini Talent | 0.175*** | 0.179*** | 0.181*** | 0.0341 |
| Concentration | (0.0194) | (0.0200) | (0.0276) | (0.0241) |
|  |  |  |  |  |
| Year Dummies | No | Yes | Yes | Yes |
|  |  |  |  |  |
| Country Dummies | No | No | Yes | Yes |
|  |  |  |  |  |
| Division Dummies | No | No | No | Yes |
|  |  |  |  |  |
| EXP | 0.0129 | 0.0169 | 0.00153 | 0.00313 |
|  | (0.0112) | (0.00987) | (0.00953) | (0.00942) |
| CONT | 0.0437*** | 0.0442*** | 0.0195*** | 0.0250*** |
|  | (0.00624) | (0.00598) | (0.00288) | (0.00253) |
| Constant | 0.0966*** | 0.0873*** | 0.0980*** | 0.145*** |
|  | (0.00808) | (0.00567) | (0.00974) | (0.00771) |
| *N* | 285 | 285 | 285 | 285 |
| *Adj R2*100* | 34.2 | 36.5 | 53.5 | 62.9 |

Standard errors (clustered at country level) in parentheses. EXP (for expansion) and CONT (for contraction) are dummy variables that take a value of one if there are more or fewer teams in the league compared to the previous season.

^*^ *p* < 0.10, ^**^ *p* < 0.05, ^***^ *p* < 0.01

**Table A3:** Estimation Results

Panel a: Theil Index

| Model | (1) | (2) | (3) | (4) |
| --- | --- | --- | --- | --- |
| Dependent Variable | Theil Index | | | |
| Gini Talent | 0.139*** | 0.142*** | 0.138*** | 0.0217 |
| Concentration | (0.0162) | (0.0161) | (0.0218) | (0.0155) |
|  |  |  |  |  |
| Year Dummies | No | Yes | Yes | Yes |
|  |  |  |  |  |
| Country Dummies | No | No | Yes | Yes |
|  |  |  |  |  |
| Division Dummies | No | No | No | Yes |
|  |  |  |  |  |
| Constant | 0.0684*** | 0.0613*** | 0.0749*** | 0.111*** |
|  | (0.00639) | (0.00455) | (0.00783) | (0.00517) |
| *N* | 285 | 285 | 285 | 285 |
| *Adj R2*100* | 27.0 | 29.2 | 47.5 | 56.5 |

Standard errors (clustered at country level) in parentheses

^*^ *p* < 0.10, ^**^ *p* < 0.05, ^***^ *p* < 0.01

Panel b: Relative deviation from the mean

| Model | (1) | (2) | (3) | (4) |
| --- | --- | --- | --- | --- |
| Dependent Variable | Relative deviation from the mean | | | |
| Gini Talent | 0.0914*** | 0.0933*** | 0.0846*** | 0.0134 |
| Concentration | (0.0110) | (0.0110) | (0.0133) | (0.0122) |
|  |  |  |  |  |
| Year Dummies | No | Yes | Yes | Yes |
|  |  |  |  |  |
| Country Dummies | No | No | Yes | Yes |
|  |  |  |  |  |
| Division Dummies | No | No | No | Yes |
|  |  |  |  |  |
| Constant | 0.0113** | 0.00459 | 0.0153*** | 0.0383*** |
|  | (0.00403) | (0.00310) | (0.00476) | (0.00407) |
| *N* | 285 | 285 | 285 | 285 |
| *Adj R2*100* | 29.1 | 31.9 | 51.1 | 59.6 |

Standard errors (clustered at country level) in parentheses

^*^ *p* < 0.10, ^**^ *p* < 0.05, ^***^ *p* < 0.01
